# Severe hypersensitivity reaction to etoposide phosphate: A case report

**DOI:** 10.1002/ccr3.2732

**Published:** 2020-08-06

**Authors:** Camille Cotteret, Julia Rousseau, Kaouther Zribi, Joel Schlatter

**Affiliations:** ^1^ Hôpital universitaire Necker–Enfants Malades APHP Pharmacie Paris France

**Keywords:** adverse drug reaction, anaphylactic shock, etoposide phosphate, hypersensitivity

## Abstract

Hypersensitivity to etoposide phosphate has rarely been documented. We report a case of severe hypersensitivity reaction to etoposide phosphate in an old man. The patient experienced anaphylactic shock and has been hospitalized in intensive care unit. Vigilance is required regarding potential severe reactions with etoposide phosphate formulation.

## Graphical abstract

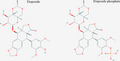

## 1 INTRODUCTION

Etoposide is a semisynthetic derivative of podophyllotoxine which induces single‐stranded DNA breaks by an interaction with DNA topoisomerase II or the formation of free radicals.^1^, ^2^, ^3^ This antineoplastic agent is used routinely in combination chemotherapy for f various neoplastic diseases such as refractory testicular cancer, small‐cell lung cancer leukemia, lymphomas, and solid tumors.^4^, ^5^ Hypersensitivity reactions to etoposide have been reported in 1%‐3% of patients characterized by hypotension, bronchospasm, urticaria, and respiratory distress.^6^, ^7^, ^8^, ^9^, ^10^ One hypothesis of hypersensitivity reactions was the presence of the polysorbate 80 diluent in which the drug is diluted.^9^ As substitution to etoposide, etoposide phosphate (Etopophos), a water soluble prodrug of etoposide that contains neither polysorbate 80 nor benzyl alcohol, has been successfully used without hypersensitivity prophylaxis.^6^, ^9^ However, a literature search revealed previous reports of etoposide phosphate hypersensitivity.^11^, ^12^, ^13^ A first case of successful treatment with etoposide base after an acute hypersensitivity reaction to etoposide phosphate has been reported by Leguay et al^12^ In this report, we present a case of patient who developed hypersensitivity reactions to etoposide phosphate.

## 2 CLINICAL REPORT

A 66‐year‐old man was diagnosed with a stage 3 enteropathy‐associated T‐cell lymphoma (EATL). He received 4 cycles of BV‐CHP (brentuximab vedotin 125‐mg, cyclophosphamide 1360‐mg, and doxorubicin 92‐mg) followed by high dose of methotrexate (5400 mg) associated with etoposide phosphate (360 mg). Immediately upon initiation of the etoposide phosphate infusion, the patient experienced a severe hypersensitivity reaction and was admitted to the intensive care unit. The patient developed an anaphylactic shock with rash, low blood pressure at 70/50 mm Hg, tachycardia, and oxygen desaturation. Immediately, subcutaneous administration of adrenalin 0.2 mg, intravenous administration of hydrocortisone hemisuccinate 50 mg, vascular filling, and oxygen therapy were initiated. The neurological, cardiovascular, and chest tests were normal with vigilant patient (gunshot wound at 15). The papillary lesions decreased rapidly with clinical positive evolution. Following this event, the patient completed the methotrexate associated with etoposide intensification using the etoposide base formulation and the BEAM regimen without complications.

## 3 DISCUSSION

Hypersensitivity reactions including anaphylaxis to etoposide are not uncommon. The mechanism involved in these reactions was assumed by several authors to be secondary to the excipient (polysorbate 80) and not etoposide itself.^11^ Polysorbate 80 consists of a mixture of fatty acid esters of sorbitol‐derived cyclic ethers and polyethylene glycol and has been documented in the immunology literature to be a type IV allergen. It induces immediate‐type non–IgE‐mediated hypersensitivity reactions via complement activation and basophile degranulation.^11^ Because etoposide phosphate is soluble in water, it is free of polysorbate 80 and therefore could be used successful after hypersensitivity to etoposide. A case report has documented an anaphylactic reaction to both etoposide phosphate and etoposide base suggesting this reaction may not be secondary to the polysorbate 80 solubilizing agent but to the etoposide itself.^11^ In rare patients, hypersensitivity to etoposide might be intrinsic to its molecular structure as opposed to the solubilizing agent employed in preparation of etoposide.^11^

We report a case of a patient who developed an immediate hypersensitivity reaction during the first infusion of etoposide phosphate and alternatively received etoposide safely. Based on available data, the proposed explanation of hypersensitivity reaction to etoposide phosphate was hypersensitivity to dextran 40, the vehicle used.^12^ Dextran was used in both vascular surgery and plastic and reconstructive surgery for its capacity to reduce platelet aggregation and to promote blood flow in the microcirculation.^15^ Severe hypersensitivity reactions to dextran have been highlighted since the 1960s.^16^, ^17^ Anaphylactic reactions induced by dextran were characterized by bronchospasm, severe hypotension, heart arrest, or death.^15^ The most probable mechanism was attributed to immune complex‐mediated reactions caused by natural antibodies reactive to dextran with complement activation and anaphylatoxin release.^17^, ^18^ In a Swedish retrospective cohort study conducted between 1975 and 1979, the incidence of severe anaphylactoid reactions due to dextran per unit of dextran 40 was reported to be 13/100 000 doses.^19^ Number of reports has declined since 1995 because of the decrease in clinical practice use.

Our clinical case shows that hypersensitivity reactions to etoposide phosphate are still present which could lead to dextran‐free formulations as an alternative.

## CONFLICT OF INTEREST

None declared.

## AUTHOR CONTRIBUTIONS

CC: collected the data of the patient and finished the writing of the manuscript. JR: collected the data of the patient. KZ: reviewed and approved the final draft. JS: wrote the first draft of the manuscript, and reviewed and approved the final draft.
